# Factors influencing client recall of contraceptive counseling at community-based distribution events in Kinshasa, Democratic Republic of the Congo

**DOI:** 10.1186/s12913-021-06796-4

**Published:** 2021-08-09

**Authors:** Rebecca E. Rosenberg, Pierre Z. Akilimali, Julie H. Hernandez, Jane T. Bertrand

**Affiliations:** 1 Avenir Health, 655 Winding Brook Dr., 4th Floor, Glastonbury, CT 06033 USA; 2 Kinshasa School of Public Health, Universite de Kinshasa, Kinshasa, Democratic Republic of the Congo; 3 Tulane School of Public Health and Tropical Medicine, 1440 Canal St., Suite 1900, New Orleans, LA 70112 USA

**Keywords:** Family planning, Community-based distribution, Quality, Sub-Saharan Africa

## Abstract

**Background:**

Clients must recall information from contraceptive counseling sessions to properly use their chosen method. Client recall in community-based settings is challenging given the public nature of these events and the presence of many potential distractions. Understanding the factors that influence client recall during community-based distribution events can guide future training of providers to improve proper use of contraceptive methods and client satisfaction.

**Methods:**

This cross-sectional study employed a convenience sample of 957 women ages 15–49 old who sought contraceptive services from community-based contraceptive distribution events in Kinshasa, Democratic Republic of the Congo, known as *Lelo PF*. Recall scores were developed by matching direct observations with client exit interviews. The association between recall and client characteristics, provider characteristics and an index for the quality of the provider-client interaction were tested using multivariate linear regression.

**Results:**

The average recall score was 67.6%. Recall scores were higher among clients who accepted methods with simpler administration procedures, such as CycleBeads (81.3%), compared to methods requiring more medically advanced administration procedures, such as DMPA-SC (56.6%) and Implanon-NXT (62.1%). This relationship held even after controlling for amount of information each client received. Status as a first-time user was associated with a 5.8 percentage point decrease in recall score (*p* = 0.002). Time since the provider’s initial family planning training and clients’ perception of the provider-client interaction were associated with higher client recall scores.

**Conclusion:**

Results of this study suggest that to improve client recall at *Lelo PF* events, future provider training should focus on how to deliver clear, specific information to clients, making sure clients feel at ease during the counseling session, and treating clients with respect. First-time family planning users and clients who select methods with more medically advanced administration procedures may require extra attention during the consultation to ensure they are able understand and remember the information. Results suggest that providers who have been offering services longer may be more effective in conveying information in a way that clients can remember. Program managers should consider requesting input from experienced providers to improve training sessions.

**Supplementary Information:**

The online version contains supplementary material available at 10.1186/s12913-021-06796-4.

## Background

Clients must recall information from contraceptive counseling sessions to be able to use their chosen method correctly. The provider-client interaction determines the accuracy and completeness of the information the client receives and may be the driving force behind what the client is able to understand, remember and successfully implement on her own [1]. Inadequate recall has been associated with poor adherence to proper contraceptive use, which may result in unintended pregnancy [2, 3]. Some of the most common errors in compliance are due to inadequate counseling, difficulty remembering instructions or both [2].

Quality of the counseling session, especially the provider’s interpersonal skills may be closely tied to client satisfaction and the clients’ ability to recall information they received. One study found quality of the provider’s interpersonal skills to have a larger impact on client outcomes than the amount of the teaching instruction [4]. In fact, clients may get overwhelmed by the amount of information they receive during a counseling session, which will hinder their ability to recall what they were told [2, 5].

There have been several studies on patients’ ability to recall information from medical visits, mostly outside of the field of reproductive health. One study assessing patient recall of health behavior discussions found that less than 50% of physician advice on diet, exercise and smoking is recalled by patients [6]. Another study exploring the amount of medical information laypeople can recall showed that, on average, clients were only able to recall 7 out of 28 items of information [5]. However, recall tends to be better during wellcare visits than ill visits, and younger, healthier patients (such as contraceptive users) tend to recall better than older, less healthy patients [6, 7]. A study examining recall in a contraceptive clinical trial found that only 23% of patients accurately recalled the pregnancy risk, but over 80% recalled being told about side effects and what those side effects were [7].

To our knowledge, this is the first study to measure client recall during community-based distribution events of family planning (FP) services. Data are from community-based contraceptive distribution events in 33 different health zones (HZs) in Kinshasa, Democratic Republic of the Congo (DRC). The province of Kinshasa is home to about 14% of the national population and contains the capital city of the DRC [8]. Contraceptive use in Kinshasa is much higher than the national average, but modern use is still low by global standards. Among women ages 15–49 in Kinshasa, 43.6% are using some form of contraception with 24.5% using a modern method [9]. Kinshasa has a large concentration of FP services due in part to a strong commitment from a variety of donor organizations and its mostly urban composition [8].

Community-based distribution is a widely accepted means of improving geographic and financial access to contraceptive services [10–14]. In urban settings, such as Kinshasa, where physical access is not a major barrier to use, community-based distribution efforts are important in overcoming social, psychological and administrative barriers in accessing contraceptive methods [15]. Increased visibility of modern contraceptive methods in the community, and easily accessible accurate information on their risks and benefits may help to eliminate some of the taboos associated with modern contraceptive use. However, client recall in these settings is especially challenging given the public nature of community-based distribution events and the presence of many potential distractions.

Community-based distribution of FP services is included in the DRC’s *National Multisectoral Strategic Plan for Family Planning (2014–2020)* and in recent years, a variety of approaches to service delivery have been introduced in Kinshasa, including community-based distribution of highly desired methods and youth-focused programs.

### Study rationale

Little is known about client recall of contraceptive counseling, particularly at community-based distribution events. Community-based FP distribution programs are widespread throughout the developing world and there is a large body of literature citing its effectiveness in addressing issues related to geographic and financial access, advancing equity in accessibility of services, enhancing contraceptive knowledge, and increasing contraceptive use and continuation rates. Community-based FP distribution is particularly important for reaching underserved populations, so ensuring quality and effectiveness of these distribution methods is crucial for achieving more equitable FP programs.

Assessing client recall, including how it is associated with the quality of the client-provider interaction and determining which provider and client characteristics may influence a client’s ability to recall information have important implications for program managers. Understanding the provider-level factors that influence client recall can help in tailoring provider trainings to enhance the skills that are associated with higher client recall. Distinguishing the patient characteristics associated with lower recall may aid providers in determining which clients to target with further intervention or more specialized counseling to avoid misuse of the method and unintended pregnancy. Ultimately, improving client recall will improve proper adherence to correct contraceptive use, which may improve client satisfaction with their chose method, enhance method continuation and reduce unintended pregnancies.

## Methods

### Study setting

In an effort to increase modern contraceptive use in Kinshasa, Tulane University undertook AcQual III beginning in July 2018. AcQual stands for “access and quality,” and the AcQual III model combines four categories of service providers (nursing students, recent nursing school graduates, resident community-based distributors [CBDs] and facility-based clinical providers) into a single coordinated service delivery model, designed to significantly increase access and improve quality of services in Kinshasa. Resident CBDs are community health works without formal clinical training who were trained for the specific purpose of contraceptive distribution. Community-based service delivery under the AcQual model occurs during FP campaign days known as *Lelo PF* as well as during home visits and in some cases at the homes of the CBDs themselves. *Lelo PF* events are coordinated by one of three different partners, depending on the health zone (HZ): *Association pour le Bien-Être Familial* (ABEF), *Santé Rural* (SANRU) or *Programme Militaire de Sante de Reproduction* (PMSR). This study focuses on the *Lelo FP* component of the AcQual model. Under this model, services take place in large multi-room tents. The tents clearly advertise the event and provide some degree of privacy from passersby. Service delivery provided in nearby facilities and at client and/or provider homes is not included in this study.

We employed a cross-sectional study design to capture information on client characteristics, provider characteristics, client recall of contraceptive counseling and quality of the provider-client interaction during *Lelo PF* events. Provider-client consultations were observed for an average of 12 providers (7 nursing school graduates and 5 resident CBDs) in 33 different HZs, followed by in-person client exit interviews (CEIs) and provider interviews.

### Study instruments and ethical considerations

Both CEIs and clinical observations (COs) were used to capture individual quality items. Twelve clinical professionals (i.e., doctors, nurses) were trained as observers in the study design, research ethics and administration of the CO. It measured technically correct counseling and method administration (including pregnancy and eligibility screening, explanation of use and possible side effects), correct medical procedure and infection prevention practices for sub-cutaneous Depot-medroxyprogesterone acetate (DMPA-SC) injection and Implanon-NXT insertion and removal, referral to fixed facilities for Jadelle, intrauterine devices (IUDs), sterilization and management of side effects, and respectful treatment of the client by the provider. Fifteen experienced interviewers were trained in the study design, research ethics and administration of the CEI. The CEI measured (in addition to socio-demographic data and FP history for each client) informed choice on the part of the client, counseling received on the full range of methods, respectful treatment of the client by the provider, and satisfaction with the information and services received. Clinical observers and interviewers were both male and female. Interviews were conducted in a separate vicinity from the service provision, but given the nature of community-based events, participants were afforded auditory privacy but not always visual privacy during the face-to-face interviews. CEIs and COs were pilot tested before the start of data collection.

Provider interviews were also conducted as part of this study. In addition to demographic characteristics, the provider interview measured correct knowledge of key factors related to contraceptive methods, willingness to serve all clients regardless of age and marital status, referrals to fixed facilities for Jadelle, IUDs, sterilization or management of side effects, perceptions of training received, frequency of stockouts and frequency of participation in community-based distribution events.

All participants in this study received a consent form, which included information about the study, why it was being performed, risks and benefits of participating, and confidentiality procedures. Clients and providers were made aware that participation in the study was voluntary and written consent was obtained from every participant. To reduce social desirability bias, efforts were made to visually distinguish the research team from providers (i.e., providers wore tan vests and logoed shirts, and the research team wore business casual attire), and clients were assured that their responses would remain confidential and would not affect future services.

### Data collection procedure and variables of interest

CEIs, COs and provider interviews were matched using provider ID numbers. Each provider was observed during three provider-client interactions. Client ID numbers consisted of the provider’s ID number followed by “1,” “2,” or “3,” to indicate whether the client was the first, second or third client to be observed with that provider. This numbering system allowed us to easily match clients and providers. On average, there were 12 providers at each of the 33 *Lelo PF* events (seven resident CBDs and five nursing graduates). With three observations per provider, we were left with a convenience sample of 1179 provider-client interactions. There were two observations with incomplete data, and three observations with incorrect client ID numbers. These cases were dropped from the study. For this analysis, the nine clients who received an implant removal at the community-based event were also excluded from the sample. The survey instruments included skip-patterns for clients seeking implant removals, so they were asked a slightly different set of questions than clients who did not receive an implant removal. In addition, their experience (e.g., amount of time spent at the *Lelo PF*, pain or anxiety associated with the removal) may have impacted their perceptions of quality in ways that the other clients did not experience. After these restrictions were applied, the total sample was 1165 provider-client interactions.

Observers were trained to listen for specific information items for each method and used a checklist to indicate whether the provider mentioned each item. During the CEI, clients were asked whether they were told these same information items for their chosen method. It should be emphasized here that clients were not expected to spontaneously volunteer all the information items they remembered. Rather, clients were prompted with each information item during the CEI. For example, clients were asked “Did the provider tell you to take the pill every day?”, rather than “What did the provider tell you about pills?” Clients may have had an easier time recalling information after being prompted than they might have if they were just asked to recall everything they were told.

The provider characteristics of interest included provider’s age, sex, educational attainment, marital status, number of living children, employment outside of CBD activities, provider type (whether the provider was a recent nursing graduate or a resident CBD worker), implementing partner (each *Lelo PF* is implemented by one of three partners: SANRU, ABEF, PMSR), the number of years since the provider received their initial FP training (ranging from 1 to 4 years), and provider FP knowledge. Provider knowledge was an additive index measuring the provider’s knowledge about different contraceptive methods, ranging from 0 to 9. A list of the questions included in the provider knowledge index can be found in Additional file 1: Table A.1.

### Analysis

We were interested in measuring client recall at the *Lelo PF* events and determining the characteristics that might impact a client’s likelihood of recalling the information she received during her consultation.

Recall scores were generated by summing the number of information points the client remembered being told, dividing by the number of information items recorded during the CO and multiplying by 100. According to the CO, 36 clients were not told any of the information items, so these were coded as missing.

We examined the association between client recall scores and different client and provider characteristics. Client characteristics included educational attainment, marital status, number of living children, employment status, chosen method, whether the client was a first-time FP user, whether the client attended a group talk before the individual counseling session and amount of information the client received. Only four clients selected condoms. Due to small cell sizes, results for condom acceptors were not meaningful and were therefore not displayed in the results. We generated a continuous additive index to control for amount of information each client received. Ranging from 0 to 31, this variable included method-specific information items the client was told, as well as information given about possible changes in menstruation and potential side effects. To be clear, this variable included all the information the client was told, not just the information related to her chosen method. For example, if a client was counseled on four different methods, each with three or four different information items, all those information items were counted, regardless of the method the client chose at the end of the session.

In addition to the client and provider characteristics, we also tested the association between client recall scores and an additive index, ranging from 0 to 5 measuring the quality of the provider-client interaction. This index included the following five binary variables: whether the provider was respectful toward the client, whether the provider refrained from commenting on the age of the client, whether the provider refrained from commenting on the marital status of the client, whether the provider avoided pressuring the client to choose a specific method, and whether the provider ensured the privacy of the consultation. Because these five items were asked in both the CEI and the CO, we created two different indices to measure the quality of the provider-client interaction: one from the client’s perspective that drew the five variables from the CEI, and one from an observer’s perspective that drew the five variables from the CO.

## Results

Because of the way recall scores were generated: (number of information points remembered divided by the number of information points recorded in the CO, multiplied by 100), we expected scores to range from 0 to 100%. However, the recall score initially ranged from 0 to 400%, with a mean value of 85.3%. Any score above 100% means the client “remembered” more than she was told during the counseling session. To make this variable a more accurate measure of recall at the *Lelo PF* events, the 208 observations with a recall score greater than 100% were dropped from the analysis, leaving a sample size of 957. A breakdown of the client characteristics of the limited sample can be found in Additional file 1: Table A.2. Client demographic characteristics of the full sample and the limited sample were not statistically different, except with regard to the amount of information received. Average amount of information points received was 7.1 in the full sample and 7.7 among the restricted sample of clients with a recall score less than or equal to 100%. Characteristics of providers who served clients in the restricted sample did not differ from the characteristics of providers in the full sample. Further discussion on this decision and its implications are in the sections below.

Table 1 shows demographic characteristics of the clients who had a recall score less than or equal to 100%. Over a third of all clients were under the age of 25 (40.5%) and almost half had never been married (46.9%). The majority (55.8%) had completed primary education, and only 3.4% completed higher education. Most had at least one child, with over half (53.6%) having one to three living children. Over one-third of clients were first-time FP users (37.6%), almost half had attended a group counseling session before the one-on-one counseling session (47.3%), and the average amount of information received during a consultation was about 8 information items.

**Table 1:** Demographic characteristics of clients with recall scores ≤100

	***N*** = 957	
Variable	n	%
**Age**		
15–24	388	40.5
25–34	382	39.9
35–49	187	19.5
**Educational attainment**		
None	60	6.3
Primary	533	55.8
Secondary	331	34.6
Higher	32	3.4
**Marital status**		
Never married	449	46.9
Married/in union	459	48.0
Divorced	41	4.3
Widow	8	0.8
**Number of living children**		
0	188	19.6
1–3	513	53.6
4–6	229	23.9
7+	27	2.8
**Employment status**		
No job	498	52.1
In-kind payment	442	46.2
Cash job	16	1.7
**Amount of information received**		
Mean points of information	7.7	–
**First-time FP user**	360	37.6
**Attended group counseling**	452	47.3

Demographic characteristics of providers are displayed in Table 2. About 40% of providers were recent nursing school graduates. Almost one-third of all providers were male, and the average age of providers was 37.1, with a range from 18 to 78. For almost half of all providers (42.5%), it had been 4 years since they received their FP training. Half of all providers (50.6%) participated in CBD activities at least once a week, but 15.3% only participated in CBD activities at campaign events or less than once a month. The average knowledge score was 6.6.

**Table 2:** Demographic characteristics of providers who participated in the study (*N* = 393)

Variable	n	%
**Provider type**		
Nursing graduate	158	40.2
Resident CBD	235	59.8
**Sex**		
Male	129	32.8
Female	264	67.8
**Average age**	393	37.1
**Educational attainment**		
None	1	0.3
Primary	37	9.4
Secondary	144	36.6
Higher	211	53.7
**Marital status**		
Never married	198	50.4
Married/in union	146	37.2
Divorced	21	5.3
Widow	27	6.9
No response	1	0.3
**Number of living children**		
0	154	39.2
1–3	105	26.7
4–6	100	25.4
7+	34	8.7
**Other employment**		
No job	189	48.1
In-kind job	4	1.0
Cash job	200	50.9
**Years since training**		
One year	84	21.4
Two years	73	18.6
Three years	69	17.6
Four years	167	42.5
**Implementing partner**		
ABEF	216	55.0
SANRU	165	42.0
PMSR	12	3.1
**Frequency participating in CBD activities**		
Only at campaigns	31	7.9
At least once a year	29	7.4
At least once a month	134	34.1
At least once a week	199	50.6
**Knowledge score**	6.6	–

The breakdown of recall scores for individual information items by method appears in Table 3. The average recall score for the entire sample was 67.6% with method-specific recall scores ranging from 56.6% for DMPA-SC acceptors to 81.3% for CycleBeads acceptors.

**Table 3:** Client recall scores by chosen method

Information item	Recall score
**Pill acceptors (*N*** **= 119)**	67.1
**CycleBead acceptors (*N*** **= 272)**	81.3
**Emergency contraception acceptors (*N*** **= 85)**	70.0
**DMPA-SC acceptors (*N*** **= 235)**	56.6
**Implanon-NXT acceptors (*N*** **= 243)**	62.1
**Average recall score for entire sample (*N*** **= 957)**	**67.6**

Next, the bivariate relationships between client characteristics and recall scores were tested (Table 4). Each additional information item reduced a client’s recall score by about 1.3 percentage points and being a first-time FP user reduced client recall scores by about 6 percentage points. Compared to no education, having achieved higher education was associated with a 13.4 percentage point increase in client recall score. Attending a group counseling session and the quality of the provider-client interaction from the client’s perspective (i.e., the “quality of the provider-client interaction” index in which the five variables were drawn from the CEI) were also associated with higher client recall scores (9.6 percentage points and 4.7 percentage points, respectively). When looking at the categorical variable for method choice, CycleBeads were chosen as the reference category. Recall scores were highest among CycleBeads acceptors and we felt this would make for a more straightforward interpretation of the results. In the bivariate regression between client recall scores and chosen method, clients who selected methods other than CycleBeads had statistically significantly lower client recall scores. Recall scores ranged from 11.3 percentage points lower (than CycleBeads acceptors) for EC acceptors to 24.7 percentage points lower (than CycleBeads acceptors) for DMPA-SC acceptors.

**Table 4:** Results of bivariate linear regressions between recall scores and client characteristics (*N* = 957)

Variable	Coefficient	***p***-value
**Age**	−0.01	0.950
**Educational attainment**		
None (ref)		
Primary	7.01	0.074
Secondary	4.79	0.236
Higher	13.41*	0.034
**Marital status**		
Never married (ref)		
Married/in union	−2.44	0.202
Divorced	−8.03	0.088
Widow	−0.35	0.973
**Number of living children**	−0.73	0.128
**Employment status**		
No job (ref)		
Job	2.38	0.203
**Method type**		
CycleBeads (ref)		
Pills	−14.12***	0.000
EC	−11.29**	0.001
DMPA-SC	−24.67***	0.000
Implanon-NXT	−19.19***	0.000
**Amount of information received**	−1.29***	0.000
**First-time FP user**		
No (ref)		
Yes	−5.96**	0.002
**Attended group counseling**		
No (ref)		
Yes	9.63***	0.000
**Provider-client interaction (client perspective)**	4.70*	0.010

*p < 0.05 **p < 0.01 ***p < 0.001

Bivariate associations between client recall scores and different provider characteristics are shown in Table 5. Provider age, number of living children, time since initial FP training, and FP knowledge score were all positively associated with client recall scores. In other words, older providers, providers with more living children, more experienced providers, and those with higher knowledge scores were all associated with higher recall scores. Client recall scores were higher for providers who were ever married compared to providers who were never married. Recall scores were also higher among providers who had a job (either a cash job or an in-kind payment job) outside of CBD work compared to providers who had no other employment. Resident CBDs were associated with recall scores that were almost 12 points higher compared to nursing graduates. Quality of the provider-client interaction from the observer’s perspective (i.e., the “quality of the provider-client interaction” index in which the five variables were drawn from the CO) was not statistically significantly associated with client recall scores.

**Table 5:** Results of bivariate linear regressions between recall scores and provider characteristics (*N* = 957)

Variable	Coefficient	***p***-value
**Age**	0.29***	0.000
**Sex**		
Female (ref)		
Male	−0.89	0.653
**Educational attainment**		
None (ref)		
Primary	4.12	0.886
Secondary	5.51	0.848
Higher	−2.50	0.930
**Marital status**		
Never married (ref)		
Married/in union	4.41*	0.028
Divorced	0.29	0.946
Widow	8.70*	0.020
**Number of living children**	1.03**	0.003
**Other employment**		
No job (ref)		
Job	4.15*	0.026
**Provider type**		
Nursing graduate (ref)		
Resident CBD	11.71***	0.000
**Implementing partner**		
ABEF (ref)		
SANRU	−1.73	0.365
PMSR	−7.42	0.200
**Time since training**	3.05***	0.000
**CBD frequency**		
Only at campaigns (ref)		
At least once a year	0.84	0.864
At least once a month	5.80	0.098
At least once a week	14.36***	0.000
**Knowledge score**	2.66***	0.000
**Provider-client interaction (observation)**	−0.20	0.928

*p < 0.05 **p < 0.01 ***p < 0.001

Based on the results of the bivariate relationships presented in Tables 4 and 5, correlations between statistically significant variables were tested to determine which variables to include in the final model. Provider marital status and number of living children were both highly correlated with provider age and type but were less statistically significantly associated with client recall, so they were dropped from the final model. To account for the structure of the data, standard errors were clustered at the provider level.

Results of the multivariate linear regressions are displayed in Table 6. Model 1 shows multivariate associations between client characteristics and client recall score. Attending a group counseling session before the individual counseling session and quality of the provider-client interaction from the client’s perspective were both positively associated with client recall scores. Amount of information received and being a first-time user were both associated with lower recall scores. Again, we see that compared to CycleBeads acceptors, clients who selected different methods tended to have lower recall scores.

**Table 6:** Results of multiple linear regressions between recall scores and client characteristics (Model 1), recall scores and provider characteristics (Model 2), and recall scores and all covariates (Model 3) (*N* = 957)

Variable	Model 1		Model 2		Model 3	
	coefficient	***p***-value	coefficient	***p***-value	coefficient	***p***-value
**Amount of information received**	−0.84***	0.000			−0.79***	0.000
**First-time FP user**						
No (ref)						
Yes	−6.51**	0.001			−5.82**	0.002
**Attended group talk**						
No (ref)						
Yes	4.71*	0.026			3.79	0.070
**Method type**						
Cyclebeads (ref)						
Pills	−13.09***	0.000			−12.88***	0.000
EC	−11.19**	0.002			−10.55**	0.004
DMPA-SC	−21.24***	0.000			−19.10***	0.000
Implanon-NXT	−16.33***	0.000			−11.12**	0.003
**Client’s educational attainment**						
None (ref)						
Primary	4.78	0.209			4.47	0.239
Secondary	0.58	0.886			0.46	0.908
Higher	3.71	0.551			4.40	0.481
**Provider-client interaction (client perspective)**	4.74**	0.009			4.16*	0.027
**Provider’s outside employment**						
No job (ref)						
Job			−1.29	0.620	−1.40	0.558
**Provider age**			−0.05	0.688	−0.50	0.686
**Provider type**						
Nursing graduate (ref)						
Resident CBD			10.71**	0.006	6.00	0.137
**CBD frequency**						
Only at campaigns (ref)						
At least once a year			−0.37	0.952	−0.51	0.929
At least once a month			4.59	0.347	2.03	0.655
At least once a week			9.28	0.060	4.08	0.369
**Time since training**			2.75**	0.006	2.43*	0.010
**Knowledge score**			1.40	0.152	0.99	0.269

*p < 0.05 **p < 0.01 ***p < 0.001

The associations between client recall and provider characteristics are shown in Model 2 of Table 6. Resident CBDs were associated with recall scores about 11 percentage points higher than nursing school graduates, and each additional year since provider FP training was associated with an increase in recall scores of about 2.8 percentage points. However, when controlling for other provider characteristics, provider age, employment outside of CBD activities, frequency of participation in CBD activities, and provider FP knowledge were no longer associated with client recall scores.

Finally, the results of the full model with both client and provider characteristics are displayed in Model 3 of Table 6. After controlling for client and provider characteristics, five variables were still associated with client recall score. Quality of the provider-client interaction from the client’s perspective and time since the provider received their initial FP training were both positively associated with client recall scores. The three remaining statistically significant variables were all negatively associated with client recall scores (amount of information received, first-time FP user, and chosen method). Each additional piece of information clients received was associated with a 0.8 percentage point reduction in recall. Recall scores for first-time users were about six percentage points lower than clients who were repeat users. After controlling for provider and client characteristics, compared to CycleBeads acceptors, all other methods were associated with statistically significantly lower client recall scores – ranging from 10.6 percentage points lower for EC acceptors to 19.1 percentage points lower for DMPA-SC acceptors. In other words, recall scores were highest among CycleBeads acceptors. Attending a group counseling session before the one-on-one counseling session and provider type were not significantly associated with recall scores in the full model.

## Discussion

On average, clients were able to recall just over two-thirds of the information items they received. The amount of information a client was given was negatively associated with recall, but it had less of an effect on recall when client, provider and quality characteristics entered the model. This finding suggests that the way in which information is delivered and the client’s unique circumstances may be just as important in determining how well a client will recall the information she receives.

First-time FP users tended to have lower recall compared to previous users. Many of these clients were likely hearing most of this information for the first time compared to other users who may receive the same information on a monthly or semi-annually basis (especially if they are repeat users of the same method). Attending a group counseling session before the one-on-one session was positively associated with client recall scores in the bivariate model and when client characteristics entered the model but was no longer significant when provider characteristics were introduced into the model. The additive index measuring quality of the provider-client interaction from the observer’s perspective (i.e., the five variables for the index were drawn from the CO) was not associated with client recall, but quality of the provider-client interaction from the client’s perspective (i.e., the five variables for the index were drawn from the CEI) was associated with recall scores that were about 4 percentage points higher for each additional point on the additive index. The important role that a provider’s interpersonal skills, especially sensitivity to feelings, plays in improving client recall has also been noted in the existing literature [1, 3, 4]. Developing a trusting client-provider relationship which considers the client’s emotions and preferences has been shown to improve contraceptive counseling outcomes, such as continuation and method adherence [1, 3]. In fact, Bartlett et al. found that the quality of providers’ interpersonal skills was more important in influencing patient outcomes than the amount of instruction [4]. It is interesting that quality of the counseling session from the clients’ perspective was associated with client recall scores but quality from the observers’ perspective was not. Because the interpersonal aspect of the provider-client interaction is so subjective and unique to each individual, it may be the case that outside observers are not able to measure it in a way that reflects the clients’ true feelings.

Time since the provider received their initial FP training was also positively associated with recall. Providers who have had more time to hone their skills by working with actual clients may be able to provide more effective consultations than providers who are fresh out of training. For example, as providers become more comfortable with the technical information they are expected to provide, they may be able to focus more effort on fostering positive interpersonal relationships with clients. A review of the best practices for contraceptive counseling highlights the importance of developing close personal relationships, building trust and optimizing decision making [3]. Research suggests clients value a friendly intimate relationship with their providers and that contraceptive uptake is positively impacted when providers are perceived as trustworthy [3].

Type of provider (nursing graduate vs. resident CBD) was significantly associated with client recall in the bivariate model and the model with provider characteristics but was no longer significant when client characteristics entered the model. One reason for this finding may be that provider type is closely tied to client’s chosen method. Resident CBD workers are not trained in the administration of Implanon-NXT, so all implant insertions were performed by the nursing graduates. Implanon-NXT is a more complicated method to administer than some of the other methods, so it is possible that clients counseled by nursing graduates were given more technical information than clients counseled by resident CBDs. Looking at the associations between client recall score and method chosen, we see that compared to CycleBeads, clients who chose Implanon-NXT had recall scores that were about 11 percentage points lower. CycleBeads are one of the least complicated methods to administer and does not cause potential side effects or changes in menstruation. The existing literature shows that simplicity and specificity of information shared during a counseling session is positively associated with client recall. A systematic review of interventions to improve recall of medical information concluded that simple yet specific information tended to be recalled better than general statements (e.g., “your incision will heal in 5-7 days” may be recalled better than “your incision will heal shortly”) [15]. Order of information and the structure in which information is given have also been shown to impact recall [15, 16]. To improve recall of clients who choose more technically complicated methods, providers should be trained to provide clear, specific information on side effects and changes in menstruation in a logically structured way. In addition to the type and amount of information clients receive, the client’s emotional state may also impact their ability to recall information shared during the consultation. Clients who select Implanon-NXT or DMPA-SC may be more nervous or anxious during the consultation due to fear associated with method administration (i.e., incision/insertion and injection). Existing research suggests that particularly high or low levels of anxiety can negatively impact recall [15]. To improve recall, clients selecting these methods may require special attention (e.g., more repetition) during the contraceptive counseling session.

### Limitations

The above findings must be interpreted in light of several study limitations. First, results cannot be generalized beyond the sample collected. Findings from this research are only relevant to *Lelo PF* events in the 33 HZs where data was collected. However, results from this study may inspire future research on client recall in community-based settings among a more representative sample.

Second, recall scores were calculated by comparing what was recorded during the direct observation compared to what the client reported during the client exit interview. Therefore, recall is limited to only the variables that were recorded in both instruments. There may have been other important information items that were shared during the consultation that we were unable to measure.

Because of the way recall scores were generated: (number of information points remembered divided by the number of information points recorded in the CO, multiplied by 100), we expected scores to range from 0 to 100%. However, the recall score initially ranged from 0 to 400%. In other words, 208 clients “recalled” more information than they were told during their individual counseling session at the *Lelo PF* event. We assumed this “excess recall” was the result of repeat users providing information they already knew about their chosen method, or clients who attended a group talk not being able to distinguish what information they were given during the group counseling session and what information they received during the one-on-one counseling session. However, a closer look revealed “excess recall” was just as common among first-time users and those who did not attend a group counseling session. There are several reasons for why this might be the case. First, this “excess recall” may be evidence of successful public education campaigns and social and behavioral change communications efforts in the HZs studied. Women who participated in this study may have received information about contraceptive methods though outside sources, which has led to them indicating they have received certain information even when it was not given by the provider at the *Lelo PF* event. Second, because recall was measured by comparing what was recorded by the clinical observer to what the client reported in the CEI, it is possible that the standard for whether the provider gave certain information was different between observers and clients. For example, observers may have only recorded technically correct information, whereas clients reported whether the information was shared, whether correct or not. Third, because clients were prompted with each information item (rather than having to spontaneously provide the information they were given), “excess recall” may be evidence of social desirability bias. It is possible that clients wanted to give their providers a good review by reporting that they were given all the relevant information, either through loyalty to their provider or through fear that a negative review might impact future services. To achieve a more accurate measure of recall, observations with a recall score greater than 100 were dropped from the analysis. Demographic characteristics of the full sample of clients compared to the sample of clients with recall scores less than or equal to 100 are shown in Additional file 1: Table A.2. The two samples did not differ significantly except with regard to amount of information received. Average amount of information points received among the limited sample was 7.7 compared to 7.1 for the full sample. Demographic characteristics of providers who served the full sample of clients compared to those who served the limited sample of clients with recall scores less than or equal to 100 are displayed in Additional file 1: Table A.3. There were no statistically significant differences across samples of providers. We are reasonably confident that “excess recall” was distributed randomly throughout the provider and client populations and that dropping the observations where recall scores exceeded 100% did not impact the results of this study.

It is also possible that providers may have behaved differently under direction observation, leading them to over-perform from being on their “best behavior” or to under-perform due to excess pressure or nervousness associated with being observed. In addition to this potential “Hawthorne Effect” of being observed, the observers themselves may have had their own personal experiences and biases that lead them to interpret provider behaviors differently from one another. This study did not collect demographic data on clinical observers, so we were unable to control for any personal characteristics that might have impacted the interpretation of results.

Finally, there were also several important aspects of the provider-client interaction that were not captured by the direct observation and CEI. For example, we do not know how much total information was given during each counseling session, the length of the counseling session or how the information was shared with the client. For example, some providers may have repeated the information, which has been shown to improve client recall [17].

## Conclusion

A client’s ability to retain the information received during a contraceptive counseling session directly influences her ability to properly use the method. Successful method use may be related to client satisfaction and the likelihood of continued use, which ultimately may impact unintended pregnancy rates. This research was intended to assess client recall at community-based FP distribution events in Kinshasa, DRC and how quality, client characteristics and provider characteristics might impact recall. Results have shown the importance of the client-provider interaction, particularly how the client perceives they have been treated by the provider.

This study has also revealed that first-time FP users and users who select methods requiring more medically advanced administration procedures may require extra attention during the consultation to ensure they are able understand and remember the information they are given. Results suggest that providers who have been offering services longer are more effective in conveying information in a way that clients can remember.

To improve client recall at *Lelo PF* events and other similar community-based distribution events, provider training should focus on building trust and treating clients with respect. Future trainings should also emphasize the importance of providing clear, specific information in a logically structured way. Additional research may be needed to test the most effective way to present information during community-based FP distribution events. Finally, program managers should consider requesting input and/or involvement from experienced providers during training sessions so they may impart their learned experiences to newer providers.

## Supplementary Information

**Additional file 1: Table A.1.** Questions used to generate the additive index for provider contraceptive knowledge. **Table A.2.** Client characteristics of the full sample and limited sample with recall scores ≤100%. **Table A.3.** Characteristics of providers who counseled full sample of clients and limited sample of clients with recall scores ≤100.

## Data Availability

The datasets used and/or analysed during the current study are available from the corresponding author on reasonable request.

## Abbreviations

ABEF: Association pour le Bien-Être Familial
CBD: Community-based distributor
CEI: Client exit interview
CO: Clinical observation
DMPA-SC: Subcutaneous depot medroxyprogesterone acetate
DRC: Democratic Republic of the Congo
FP: Family planning
HZ: Health zone
IUD: Intrauterine device
PMSR: Programme Militaire de Sante de Reproduction
SANRU: Santé Rural
