# Supplementary material for: Factors influencing client recall of contraceptive counseling at community-based distribution events in Kinshasa, Democratic Republic of the Congo
Source: BMC Health Serv Res. 2021 Aug 9;21:784. doi: 10.1186/s12913-021-06796-4 (PMC8351144; doi:10.1186/s12913-021-06796-4)
Supplement: Supplementary file 1 — Additional file 1: Table A.1. Questions used to generate the additive index for provider contraceptive knowledge. Table A.2. Client characteristics of the full sample and limited sample with recall scores ≤100%. Table A.3. Characteristics of providers who counseled full sample of clients and limited sample of clients with recall scores ≤100. [file 12913_2021_6796_MOESM1_ESM.docx]

**Table A.1 Questions used to generate the additive index for provider contraceptive knowledge**

| **Questions** | **Response coding for additive index** |
| --- | --- |
| Can a woman who asks for a contraceptive method be pregnant if she gave birth less than three months ago? | 1- Yes  0- No |
| Can a woman who asks for a contraceptive method be pregnant if she does not frequently have sexual intercourse? | 1- Yes  0- No |
| Can a woman who asks for a contraceptive method be pregnant if she has not had sexual intercourse since her last period? | Yes  0- No |
| Can a woman who asks for a contraceptive method be pregnant if she is HIV positive? | 1- Yes  0- No |
| In general, how long after childbirth can a woman start taking combined oral contraceptive pills (if she is not breastfeeding her child)? | 0- Immediately  1- One month  0- Three months  0- Six months  0- One year |
| In general, how long after childbirth can a woman start taking progestin only pills (if she is not breastfeeding her child)? | 1- Immediately  0- One month  0- Three months  0- Six months  0- One year |
| How many times can you use the same condom? | 1- Once  0- Twice  0- Three times |
| On the CycleBead, what color are the beads that indicate the days when the woman is likely to get pregnant? | 1- White  0- Red  0- Brown  0- Other |
| How long after a woman has unprotected sex is emergency contraception still effective? | 0- 12 hours  0- One day  1- Three days  1- Five days  0- One week |
| **Total score** | **0-9** |

**Table A.2 Client characteristics of the full sample and limited sample with recall scores ≤ 100%**

|  | **Total Sample (N=1,165)** | | **Recall Sample (N=957)** | | **Immediate test of proportions** |
| --- | --- | --- | --- | --- | --- |
| **Variable** | **n** | **%** | n | **%** | **p-value** |
| **Age** |  |  |  |  |  |
| 15-24 | 478 | 41.0 | 388 | 40.5 | 0.816 |
| 25-34 | 457 | 39.2 | 382 | 39.9 | 0.743 |
| 35-49 | 230 | 19.7 | 187 | 19.5 | 0.908 |
| **Educational attainment** |  |  |  |  |  |
| None | 64 | 5.5 | 60 | 6.3 | 0.435 |
| Primary | 646 | 55.5 | 533 | 55.8 | 0.890 |
| Secondary | 408 | 35.1 | 331 | 34.6 | 0.810 |
| Higher | 46 | 4.0 | 32 | 3.4 | 0.468 |
| **Marital status** |  |  |  |  |  |
| Never married | 563 | 48.3 | 449 | 46.9 | 0.521 |
| Married/in union | 541 | 46.4 | 459 | 48.0 | 0.463 |
| Divorced | 51 | 4.4 | 41 | 4.3 | 0.911 |
| Widow | 10 | 0.9 | 8 | 0.8 | 0.803 |
| **Number of living children** |  |  |  |  |  |
| 0 | 236 | 20.3 | 188 | 19.6 | 0.688 |
| 1-3 | 626 | 53.7 | 513 | 53.6 | 0.963 |
| 4-6 | 267 | 22.9 | 229 | 23.9 | 0.588 |
| 7+ | 36 | 3.1 | 27 | 2.8 | 0.685 |
| **Employment status** |  |  |  |  |  |
| No job | 610 | 52.4 | 498 | 52.1 | 0.891 |
| In-kind payment | 17 | 1.5 | 16 | 1.7 | 0.714 |
| Cash job | 537 | 46.1 | 442 | 46.2 | 0.963 |
| **Method type** |  |  |  |  |  |
| CycleBeads | 325 | 27.9 | 272 | 28.4 | 0.799 |
| Condoms | 4 | 0.3 | 3 | 0.3 | 1.000 |
| Pills | 156 | 13.4 | 119 | 12.4 | 0.495 |
| EC | 113 | 9.7 | 85 | 8.9 | 0.529 |
| DMPA-SC | 277 | 23.8 | 235 | 24.6 | 0.668 |
| Implanon NXT | 290 | 24.9 | 243 | 25.4 | 0.792 |
| **Amount of information received** |  |  |  |  |  |
| Mean points of information | 7.1 | --- | 7.7 | --- | 0.021* |
| **First-time FP user** | 427 | 36.7 | 360 | 37.6 | 0.669 |
| **Attended group counseling** | 565 | 48.5 | 452 | 47.3 | 0.582 |

**Table A.3 Characteristics of providers who counseled full sample of clients and limited sample of clients with recall scores ≤ 100**

|  | **Total Sample (N=1,165)** | | **Recall sample (N=957)** | | **Immediate test of proportions** |
| --- | --- | --- | --- | --- | --- |
| **Variable** | **n** | **%** | **n** | **%** | **p-value** |
| **Average age** | 37.2 | --- | 37.2 | --- | 0.970 |
| **Sex** |  |  |  |  |  |
| Male | 385 | 33.1 | 319 | 33.3 | 0.922 |
| Female | 780 | 67.0 | 638 | 66.7 | 0.884 |
| **Educational attainment** |  |  |  |  |  |
| None | 3 | 0.3 | 1 | 0.1 | 0.316 |
| Primary | 111 | 9.6 | 98 | 10.3 | 0.591 |
| Secondary | 432 | 37.2 | 333 | 34.9 | 0.273 |
| Higher | 616 | 53.0 | 522 | 54.7 | 0.435 |
| **Marital status** |  |  |  |  |  |
| Never married | 582 | 50.1 | 478 | 50.1 | 1.000 |
| Married/in union | 437 | 37.6 | 359 | 37.6 | 1.000 |
| Divorced | 62 | 5.3 | 51 | 5.3 | 1.000 |
| Widow | 81 | 7.0 | 67 | 7.0 | 1.000 |
| **Number of living children** |  |  |  |  |  |
| 0 | 450 | 38.6 | 366 | 38.2 | 0.851 |
| 1-3 | 314 | 27.0 | 263 | 27.5 | 0.797 |
| 4-6 | 299 | 25.7 | 246 | 25.7 | 1.000 |
| 7+ | 102 | 8.8 | 82 | 8.6 | 0.871 |
| **Other employment** |  |  |  |  |  |
| No job | 558 | 47.9 | 466 | 48.7 | 0.714 |
| In-kind job | 12 | 1.0 | 11 | 1.2 | 0.659 |
| Cash job | 595 | 47.9 | 480 | 50.2 | 0.292 |
| **Provider type** |  |  |  |  |  |
| Nursing graduate | 458 | 39.3 | 389 | 40.7 | 0.512 |
| Resident CBD | 707 | 60.7 | 568 | 59.4 | 0.543 |
| **Implementing partner** |  |  |  |  |  |
| ABEF | 639 | 54.9 | 530 | 55.4 | 0.818 |
| SANRU | 491 | 42.2 | 401 | 41.9 | 0.889 |
| PMSR | 35 | 3.0 | 26 | 2.7 | 0.680 |
| **Years since training** |  |  |  |  |  |
| One year | 247 | 21.2 | 198 | 20.7 | 0.778 |
| Two years | 217 | 18.6 | 188 | 19.6 | 0.559 |
| Three years | 205 | 17.6 | 177 | 15.8 | 0.270 |
| Four years | 496 | 42.6 | 394 | 41.2 | 0.516 |
| **Frequency participating in CBD activities** |  |  |  |  |  |
| Only at campaigns | 93 | 8.0 | 81 | 8.5 | 0.677 |
| At least once a year | 86 | 7.4 | 56 | 5.9 | 0.170 |
| At least once a month | 397 | 34.1 | 348 | 36.4 | 0.270 |
| At least once a week | 589 | 50.6 | 472 | 49.3 | 0.552 |
| **Knowledge score** | 6.6 | --- | 6.6 | --- | 0.828 |
